# A comparison of sLASER and MEGA-sLASER using simultaneous interleaved acquisition for measuring GABA in the human brain at 7T

**DOI:** 10.1371/journal.pone.0223702

**Published:** 2019-10-11

**Authors:** Donghyun Hong, Seyedmorteza Rohani Rankouhi, Jan-Willem Thielen, Jack J. A. van Asten, David G. Norris

**Affiliations:** 1 Erwin L. Hahn Institute for Magnetic Resonance Imaging, University of Duisburg-Essen, Essen, Germany; 2 Department for Psychiatry and Psychotherapy, Faculty of Medicine, University of Duisburg-Essen, Essen, Germany; 3 Department of Radiology and Nuclear Medicine, Radboud University Medical Center, Nijmegen, Netherlands; 4 Donders Institute for Brain, Cognition and Behavior, Radboud University, Nijmegen, Netherlands; Linköping University, SWEDEN

## Abstract

γ-Aminobutyric acid (GABA), the major inhibitory neurotransmitter, is challenging to measure using proton spectroscopy due to its relatively low concentration, J-coupling and overlapping signals from other metabolites. Currently, the prevalent methods for detecting GABA at ultrahigh field strengths (≥ 7 T) are GABA-editing and model fitting of non-editing single voxel spectra. These two acquisition approaches have their own advantages: the GABA editing approach directly measures the GABA resonance at 3 ppm, whereas the fitting approach on the non-editing spectrum allows the detection of multiple metabolites, and has an SNR advantage over longer echo time (TE) acquisitions. This study aims to compare these approaches for estimating GABA at 7 T. We use an interleaved sequence of semi-LASER (sLASER: TE = 38 ms) and MEGA-sLASER (TE = 80 ms). This simultaneous interleaved acquisition minimizes the differential effect of extraneous factors, and enables an accurate comparison of the two acquisition methods. Spectra were acquired with an 8 ml isotropic voxel at six different brain regions: anterior-cingulate cortex, dorsolateral-prefrontal cortex, motor cortex, occipital cortex, posterior cingulate cortex, and precuneus. Spectral fitting with LCModel quantified the GABA to total Cr (tCr: Creatine + Phosphocreatine) concentration ratio. After correcting the T_2_ relaxation time variation, GABA/tCr ratios were similar between the two acquisition approaches. GABA editing showed smaller spectral fitting error according to Cramér–Rao lower bound than the sLASER approach for all regions examined. We conclude that both acquisition methods show similar accuracy but the precision of the MEGA-editing approach is higher for GABA measurement. In addition, the 2.28 ppm GABA resonance was found to be important for estimating GABA concentration without macromolecule contamination in the GABA-edited acquisition, when utilizing spectral fitting with LCModel.

## Introduction

γ-Aminobutyric acid (GABA), the major inhibitory neurotransmitter, is present at about one millimolar concentration in the human brain [1]. GABA plays an important role as a potential biomarker in neurological and neuropsychiatric disorders such as cancer [2], multiple sclerosis [3], Alzheimer’s disease [4], epilepsy [5], schizophrenia [6], and autism [7].

Existing *in-vivo* approaches for measuring GABA include positron emission tomography (PET) [8], single photon emission tomography (SPECT) [9], proton magnetic resonance spectroscopy (^1^H MRS) [10] and chemical exchange saturation transfer (CEST) [11, 12]. Among these, MRS at ultrahigh filed (UHF ≥ 7 T) is widely used due to high sensitivity and specificity for measuring GABA [13]. Nevertheless, GABA MRS is still challenging by reason of its low concentration and resonances overlapping with higher concentration signals [14]. Previously, various acquisition strategies for proton MRS have been proposed for measuring GABA, such as J-editing [15] (e.g. MEGA-PRESS (MEsher-GArwood Point RESolved Spectroscopy) [16, 17]) and double quantum filters [18].

The MEGA-editing method [14] is the most commonly used approach. A MEGA pulse is used to distinguish the GABA signal at 3 ppm from the overlapping creatine signal, based on the J difference editing approach [16, 17]. MEGA-editing combines conventional localization techniques with one or more additional frequency selective editing pulses, which invert the signal at 1.9 ppm. As the GABA resonance at 3 ppm signal is coupled to the 1.9 ppm GABA resonance, the difference between the signals obtained with and without the editing pulses should give an unambiguous GABA signal at 3 ppm. However, the MEGA-editing method also has disadvantages. It theoretically works best at certain echo times (TEs), when the GABA is refocused in anti-phase to the signal obtained without editing pulses (TE = (2n-1) / 2J, where J is 7.35 Hz, n = 1, 2, 3…). Therefore, the TE for GABA editing is largely standardized at 68 ms as the next possible TE beyond 68 ms (204 ms) would imply a significant T_2_ decay of the signal. At 7 T, it is quite challenging to implement the MEGA-semi-LASER(MEGA-sLASER) sequence with TE = 68 ms due to the lack of time to insert all pulses within 68ms. Therefore, longer TEs have frequently been used (e.g., 72 ms [19] and 74 ms [6, 20, 21]. However, Edden, *et al*. [22] reported that a TE of 80 ms showed a reduction in co-edited macromolecule (MM) compared to TE 68 ms even though the GABA SNR is reduced by approximately 7% in comparison with that of TE = 68 ms due to the combined but opposing effects of increased editing efficiency and greater T_2_-relaxation. In addition, motion, respiration, and frequency drift induce experimental instabilities that may result in subtraction artifacts [23].

Another extensively used GABA measurement method is to obtain a non-edited spectrum, and measure the GABA signals using spectral fitting via LCModel [24, 25]. As these data are normally acquired at a shorter TE, the acquired spectrum is superior to the edited spectrum in terms of sensitivity. In addition, not only GABA but also all metabolites are quantified simultaneously. However, a spectral fitting procedure is required in order to measure metabolite concentrations. Previously, a number of studies have shown the feasibility of GABA detection in combination with LCModel analysis at different field strength: 3 T [26, 27], 4 T [28] and 7 T [29, 30]. Even though GABA is detectable at various field strengths, it has also been reported that spectra that are acquired at higher field strength show superior fitting precision due to the high spectral dispersion, better SNR and simplified spectra [31].

Given these two leading but different GABA measurement techniques: GABA-editing and non-editing acquisitions, there have been various attempts to assess their relative merits. Terpstra, *et al*. [32] compared STEAM (Stimulated Echo Acquisition Mode [33–35], TE = 5 ms) with MEGA-PRESS (TE = 68 ms) at 4 T in a validation study measuring glutathione (GSH). The concentrations of GSH measured with these two methods in that study were similar, suggesting a similar accuracy of the two techniques for those conditions. Sanaie Nezhad, *et al*. [36] compared the accuracy and sensitivity of PRESS (Point RESolved Spectroscopy [37, 38], TE = 35 ms) and MEGA-PRESS (TE = 130 ms) at 3 T to measure GSH, and found that PRESS is not an accurate and reliable method to measure GSH *in vivo*. They also showed that the spectral fitting of the PRESS spectra cannot reliably quantify the concentration of GSH when the concentration is 4 mM or less. In addition, one conference proceeding by Chen, *et al*. [39] reported that the GABA to total Cr (tCr: creatine (Cr) + phosphocreatine(PCr)) ratios from occipital cortex (OC) and motor cortex (MC), measured by the MEGA sLASER (TE = 72ms) and STEAM (TE = 17ms) approaches, are comparable after T_2_ relaxation correction. However, after MM correction, GABA editing results were superior to those of the non-editing spectral acquisitions in terms of both reliability and reproducibility. Nevertheless, the authors still suspected that MM contamination and editing efficiency might have had an influence on GABA quantification.

In addition, it is widely known that the 3 ppm signal measured with the GABA-editing technique is typically contaminated by co-edited MM due to the overlapped resonance frequencies of the GABA and MM by the editing pulse, and this is generally considered to be a methodological limitation of this approach [14, 40, 41]. Therefore, this measured 3 ppm signal is commonly referred to as GABA+: GABA ‘plus’ co-edited MM [17, 42].

A standard approach to obtain (relative) GABA concentrations from non-edited single voxel spectroscopy (SVS) spectra is to use LCModel [24, 25] that fits metabolites together with additional MM, and lipid baselines. Furthermore, the GABA signal is fitted to all three methylene groups of GABA, of which the resonance at 2.28 ppm is uncontaminated by the MM signal. This should ensure that the GABA estimated in this way does not include MM. We hypothesize that if the MEGA-edited spectrum also contains a visible signal at 2.28 ppm then application of LCModel will similarly yield estimates of GABA concentration that are free of MM contamination. We can test this hypothesis by obtaining spectra from the same voxel using both acquisition techniques, to test whether the GABA concentrations obtained are the same. This study aims to compare two GABA measurement methods in combination with LCModel fitting by comparing relative GABA concentrations and corresponding spectral fitting quality at various brain regions of healthy individuals. In order to minimize the effect of external factors, a single interleaved sequence, which acquires spectra of the two measurement methods simultaneously was used. As a result, factors such as motion, shim, and B_1_ inhomogeneity should be the same for both techniques.

## Material and methods

### Sequence implementation

The MEGA method can be implemented with conventional SVS acquisition methods. It was initially implemented in a PRESS sequence [16, 17, 37, 38]. However, at ultrahigh field, PRESS loses efficiency because of higher B_1_ field inhomogeneity. In contrast, the sLASER sequence [43] offers improved performance mainly because of the use of adiabatic refocusing pulses that are insensitive to B_1_ field inhomogeneity and have high bandwidth, leading to a small chemical shift displacement error (CSDE) [44].

**Fig 1:**
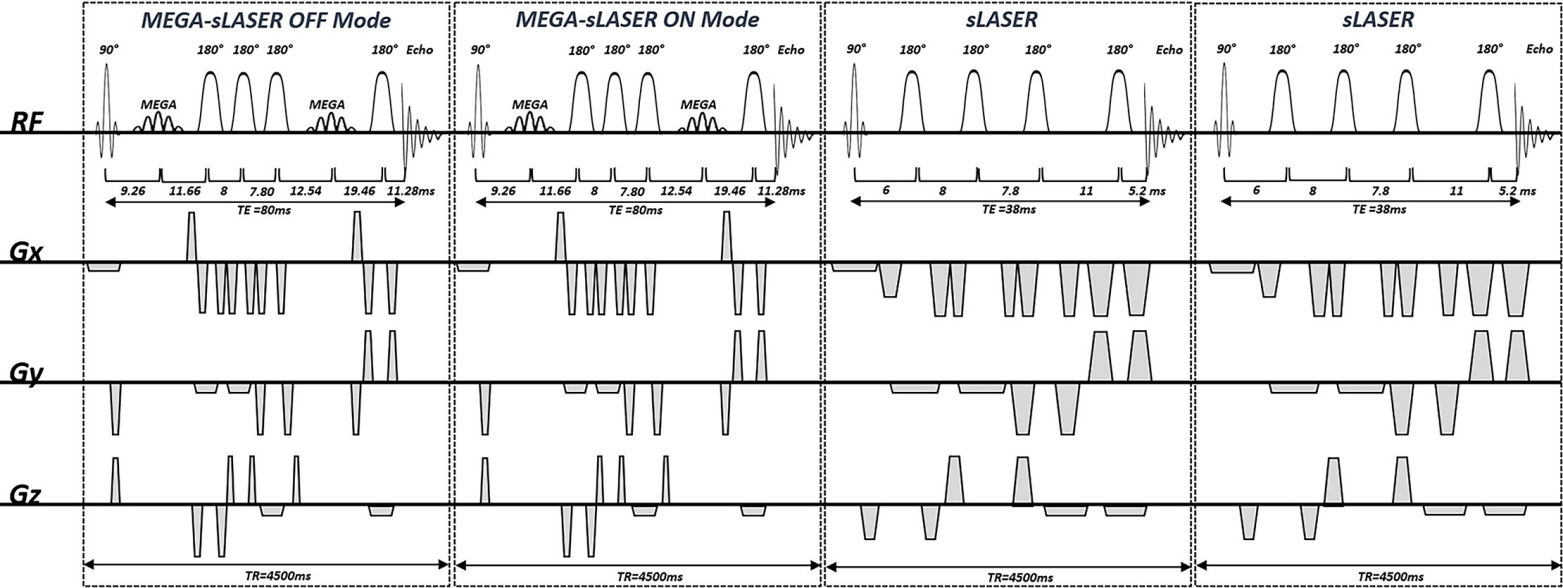
The pulse sequence diagram of the interleaved sequence. This figure shows a pulse sequence diagram of the interleaved MEGA-semi-LASER (echo time (TE) = 80 ms) and semi-LASER (TE = 38ms) sequence implemented in the current study. This sequence is composed of four sub-sequence blocks, which have identical repetition times of 4500 ms.

In this study, the sLASER sequence was used to acquire non-editing *in-vivo* spectra, and MEGA-editing was performed on the basis of the same sLASER sequence, termed MEGA-sLASER [20]. MEGA-sLASER and sLASER SVS spectra were acquired using a single interleaved sequence shown in Fig 1. It consists of the repeated application of four sub sequence blocks: a MEGA-off sLASER, a MEGA-on sLASER, and two sLASER acquisitions. The repetition time (TR) of each sub sequence block was 4500ms.

In the implementation of the sLASER sequence with TE = 38 ms, a Shinnar-Le Roux (SLR) 90° excitation pulse (duration = 3.4 ms and bandwidth = 3.5 kHz) was used for slice selection in one direction and two pairs of hyperbolic secant 180° refocusing pulses (duration = 5 ms and bandwidth = 5.3 kHz) were used for slice selection in the two other directions. The spoiler gradients had an amplitude of 25 mT/m and their duration varied between 0.8 and 2 ms. WET (Water suppression Enhanced through T1 effects) water suppression [45] with four RF pulses was used to suppress the water signal. This has a lower sensitivity to B_1_ variations compared with the three RF pulses WET water suppression. The water suppression block was placed before the localization block of the sequence and is not depicted in the sequence diagram. TE of 38 ms for this sequence is a slightly conservative choice to ensure elimination of unwanted signals and but comparable to those of other sLASER studies at the same field strength; 24 ms [46], 25 ms [47], 28 ms [6], 32 ms [31] and 36ms [48].

MEGA-sLASER was implemented with a TE of 80 ms [22]. Localization components of this sequence were the same as for the sLASER, as described above. A pair of dual-band inversion pulses (duration = 11.52 ms and bandwidth = 133 Hz) was used for both the MEGA-editing and extra water suppression. This editing pulse inverts at 1.9 and 4.7 ppm in on-mode, and at 4.7 and 7.5 ppm in off-mode. Since additional MM suppression was not included in the acquisition sequence except a prolonged TE of 80 ms, our measured GABA signal at 3 ppm is also GABA +.

### Ethics statement

The study was conducted at the Erwin L. Hahn Institute for Magnetic Resonance Imaging, University of Duisburg-Essen, Essen, Germany. The experimental protocol was approved by the Ethics Commission of the Medical Faculty of the University Duisburg-Essen (study number 11–4898-BO). All participants provided written informed consent in accordance with the declaration of Helsinki.

### Data acquisition

12 healthy volunteers (9 male and 3 female subjects, 29.33 ± 4.49 years old) participated in this study. Experiments were performed on a 7 T whole body system (Magnetom, Siemens, Erlangen, Germany) with a 32-channel transmit/receive (TX/RX) bird-cage coil (Nova Medical, Wilmington, MA). First, T_1_ weighted images were acquired as an anatomical reference using a three-dimensional magnetization-prepared rapid gradient-echo (3D MPRAGE) [49] with the following scan parameters: 256 slices, slice thickness = 1 mm, TR = 2500 ms, TE = 1.35 ms, inversion time (TI) = 1100 ms, flip angle = 6°, field of view = 256 x 256 x 256 mm^3^, scan time ≈ 5 min. B_0_ shimming was performed using FASTESTMAP [50]. Single voxel MRS data were collected from 20 x 20 x 20 mm^3^ voxels positioned at anterior cingulate cortex (AC), dorsolateral prefrontal cortex (DLPFC), MC, OC, posterior cingulate cortex (PC), and precuneus (PRC) using the interleaved MEGA-sLASER and sLASER sequence (TR = 4500 ms, TEs = 80, 80, 38 and 38 ms, 32 averages of each sub-sequence, bandwidth = 3000 Hz, data points = 1024 and total scan time ≈ 10 min).

### Data processing

The signals from each coil element were combined by the signal weighting method [51] that the manufacturer provides by default. MEGA difference spectra were calculated with the jMRUI software package (Version 6.0, http://www.jmrui.eu) [52, 53]. The nominal spectral resolution was increased to 0.27 Hz by zero-filling for a more exact adjustment. The MEGA-edited spectra were acquired by subtracting MEGA-on and MEGA-off spectra after alignment with the 3 ppm peak by the horizontal shift along the frequency axis, to avoid subtraction artifacts [23] caused by frequency drift. After subtraction, 1 Hz line broadening was applied to remove the zero-filling induced high-frequency noise. The same levels of zero-filling and line broadening were applied to the GABA-editing and the non-editing acquisition spectra.

### Data analysis

Spectral quality was evaluated on the basis of the SNR and was measured using the 2ppm resonance of total N-acetylaspartate (NAA) and the standard deviation of the white noise area in the 7 to 9 ppm range, for both GABA-editing and non-editing acquisition spectra.

LCModel software (Version 6.3-1L, Stephen Provencher, Ontario, Canada) [24, 25] was used to estimate metabolite concentrations. The Cramér–Rao lower bound (CRLB), was taken as giving the error in the metabolite quantification (expressed in %SD) [24]. For the basis set for the LCModel analysis, parametric spectral models of alanine (Ala), aspartate (Asp), ascorbate (Asc), glycerophosphocholine (GPC), choline (Cho), phosphocholine (PCh), Cr, PCr, GABA, glucose (Glc), glutamine (Gln), glutamate (Glu), glycine (Gly), GSH, myo-inositol (mI), lactate (Lac), NAA, N-acetylaspartylglutamate (NAAG), phosphoethanolamine (PE), scyllo-inositol (Scyllo) and taurine (Tau) were simulated using the NMRSIM module included in TOPSPIN suite (Version 3.6, Bruker, Rheinstetten, Germany) with identical parameters (e.g. RF pulse profile, RF pulse timing, resonance frequency, and acquisition bandwidth) to those used for the *in-vivo* acquisition. Chemical shift and J-coupling values for each metabolite were taken from [54, 55].

For the sLASER data, the basis set for the LCModel analysis consisted of all twenty-one simulated metabolites. For the GABA editing method, six edited metabolites were modelled: GABA, Glu, Gln, NAA, NAAG, and GSH were included in the basis set. Each edited spectral model was created by subtracting a simulated MEGA-off spectrum from a simulated MEGA-on spectrum. As LCModel performs a phase correction during spectral fitting, additional phase correction steps were not applied. For MM and lipid signals, the non-parametric basis sets that LCModel provides by default were used [56]. However, one singlet peak with Lorentzian lineshape was also included in the GABA-editing basis set model the MM signal at 3 ppm, and thus avoid over-estimation of GABA due to the co-edited MM [57].

We report regional GABA concentrations as a ratio relative to tCr that was normalized across the scans. We used the tCr signal in the MEGA-off spectrum as an internal reference for the MEGA-editing approach. Previous studies used cut-off criteria of 20% [58] or 30% [59] for CRLB, our study included all GABA and Cr results to avoid CRLB thresholding leading to a bias in measuring low concentration metabolites [60]. Spectral fitting quality was compared with CRLB. In addition, absolute uncertainty was calculated by multiplying the relative CRLB by the estimated concentration [60].

As the data were acquired at different TEs, the signal concentration differences were also compensated [61] for T_2_. We assumed similar T_2_ relaxation times in GM and WM for each metabolite, and used values taken from the literature of: T_2 tCr_ = 121 ms and T_2 GABA_ = 63 ms [62] at 7 T. T_1_ weighted images of each spectroscopy voxel of interest (VOI), which were also used for spectroscopy voxel placement, were segmented using SPM12 (Wellcome Trust Centre for Neuroimaging, University College London, UK) unified segmentation routines to determine the relative proportions of GM, WM and cerebrospinal fluid (CSF) within the spectroscopy voxel. We calculated a volume fraction for each tissue component.

Regional GABA concentrations and CRLBs were also compared with a one-way ANOVA was performed using SPSS (Ver. 22, IBM, NY). The null hypothesis was that there is no difference between regions. A p-value of less than 0.05 was considered statistically significant. Bland-Altman analysis [63] as implemented in MedCalc Statistical Software v18.11.6 (MedCalc Software bvba, Ostend, Belgium) was used to compare the raw GABA concentrations (determined by LCModel analysis) acquired by the two acquisition approaches over all voxels.

Potential MM contributions in the edited 3 ppm peak of the MEGA approach were also approximated by amplitude ratios between the fitted GABA line by LCModel and the acquired GABA+ peak at 3 ppm.

## Results

**Fig 2:**
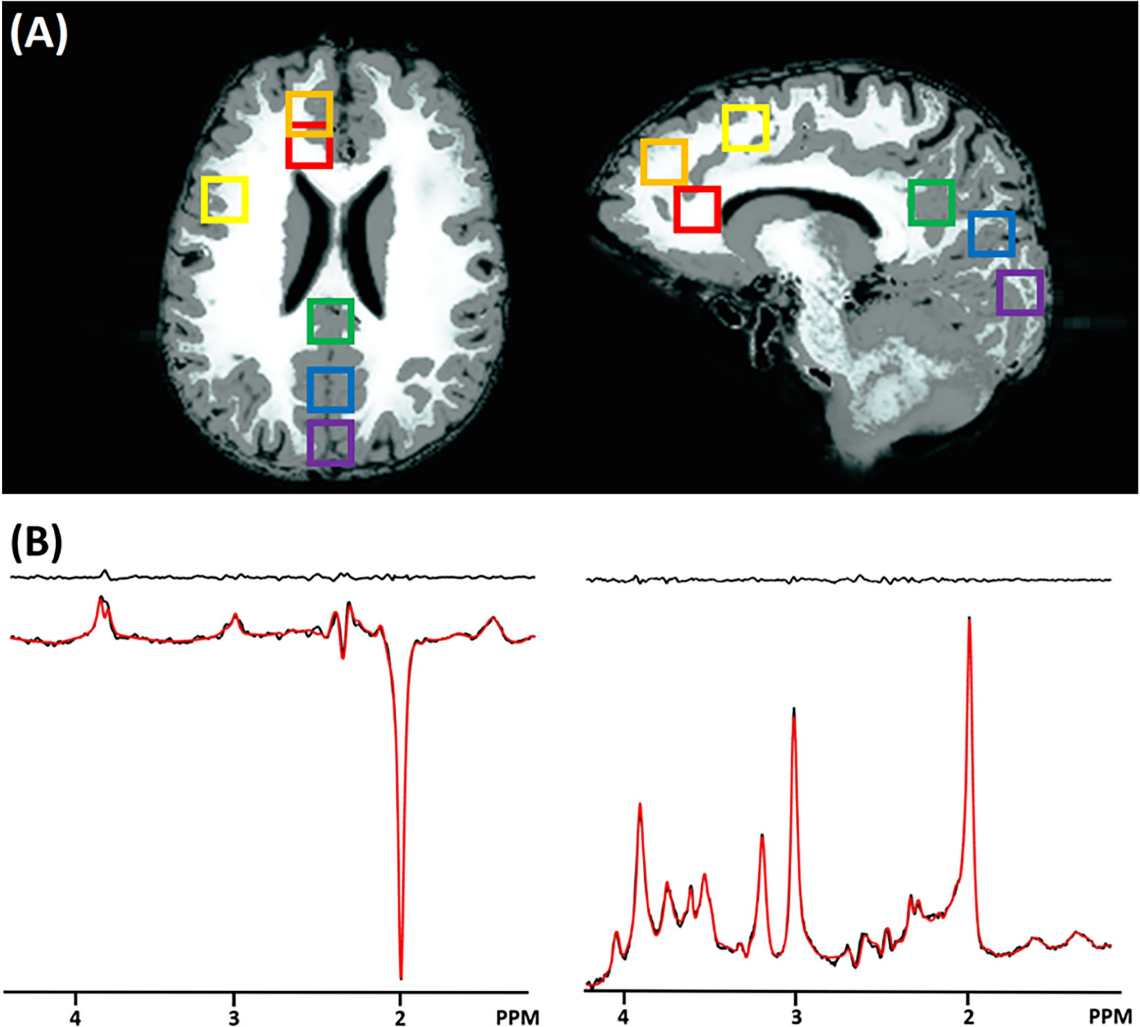
Spectroscopy voxel positions and exemplary results. (A) Six spectroscopy voxel positions: anterior cingulate cortex (AC: red), dorsolateral prefrontal cortex (DLPFC: orange), motor cortex (MC: yellow), occipital cortex (OC: purple), posterior cingulate cortex (PC: green), and precuneus (PRC: blue) on the GM (gray) and WM (white) map. (B) Example results of the LCModel analysis for the MEGA-sLASER (left) and the sLASER (right) that were acquired with the interleaved acquisition from a single voxel (PRC). The fitted line is plotted in red and residual signal (the difference between *in-vivo* and fitting lines) is plotted at the top.

Water linewidths obtained after performing 2–3 iterations with the FASTESTMAP sequence were below 14 Hz for all voxels. Fig 2A depicts locations of VOIs on the GM (gray) and WM (white) maps after T1 weighted image segmentation. Fig 2B shows examples of fitting GABA with LCModel using sLASER (top) and the MEGA-sLASER (bottom) for all six regions. Table 1 summarizes mean metabolic concentration ratios between GABA and tCr, CRLB values and absolute uncertainties for GABA and tCr at six different brain regions for the two different acquisition approaches. Measured spectral SNRs are also summarized in Table 1.

**Table 1:** Averaged SNR and LCModel results from six brain regions by the semi-LASER (sLASER) and the MEGA-sLASER approaches.

	SNR		Concentration ratio		CRLB(%)				Absolute uncertainty			
			GABA/tCr		GABA		tCr		GABA		tCr	
Technique	sLASER	MEGA-sLASER	sLASER	MEGA-sLASER	sLASER	MEGA-sLASER	sLASER	MEGA-sLASER	sLASER	MEGA-sLASER	sLASER	MEGA-sLASER
TE (ms)	38	80	38	80	38	80	38	80	38	80	38	80
AC	*511*.*80 ± 90*.*25*	*325*.*17 ± 62*.*40*	*0*.*13 ± 0*.*06*	*0*.*07 ± 0*.*03*	*41*.*20 ± 8*.*42*	*21*.*33 ± 6*.*60*	*1*.*00 ± 0*.*00*	*1*.*83 ± 0*.*67*	*0*.*17 ± 0*.*07*	*0*.*05 ± 0*.*02*	*0*.*03 ± 0*.*01*	*0*.*02 ± 0*.*01*
DLPFC	*435*.*37 ± 146*.*20*	*388*.*87 ± 93*.*90*	*0*.*22 ± 0*.*09*	*0*.*10 ± 0*.*02*	*31*.*33 ± 6*.*70*	*26*.*00 ± 6*.*68*	*1*.*33 ± 0*.*47*	*1*.*33 ± 0*.*47*	*0*.*18 ± 0*.*02*	*0*.*06 ± 0*.*02*	*0*.*05 ± 0*.*02*	*0*.*03 ± 0*.*01*
MC	*540*.*78 ± 155*.*41*	*294*.*18 ± 57*.*73*	*0*.*16 ± 0*.*03*	*0*.*11 ± 0*.*05*	*36*.*50 ± 4*.*11*	*19*.*50 ± 12*.*08*	*1*.*17 ± 0*.*37*	*1*.*17 ± 0*.*37*	*0*.*17 ± 0*.*03*	*0*.*05 ± 0*.*03*	*0*.*05 ± 0*.*03*	*0*.*04 ± 0*.*03*
OCC	*417*.*24 ± 37*.*00*	*410*.*63 ± 126*.*73*	*0*.*28 ± 0*.*03*	*0*.*19 ± 0*.*04*	*29*.*00 ± 7*.*09*	*29*.*33 ± 8*.*76*	*1*.*33 ± 0*.*47*	*1*.*17 ± 0*.*37*	*0*.*30 ± 0*.*21*	*0*.*12 ± 0*.*05*	*0*.*05 ± 0*.*03*	*0*.*03 ± 0*.*01*
PC	*441*.*11 ± 102*.*62*	*277*.*74 ± 78*.*50*	*0*.*26 ± 0*.*09*	*0*.*18 ± 0*.*04*	*31*.*83 ± 4*.*30*	*28*.*50±11*.*72*	*1*.*17 ± 0*.*37*	*1*.*33 ± 0*.*47*	*0*.*22 ± 0*.*09*	*0*.*11 ± 0*.*08*	*0*.*04 ± 0*.*02*	*0*.*03 ± 0*.*02*
PRC	*487*.*66 ± 143*.*81*	*314*.*86 ± 102*.*78*	*0*.*25 ± 0*.*05*	*0*.*18 ± 0*.*03*	*31*.*00 ± 6*.*58*	*27*.*50 ± 5*.*19*	*1*.*17 ± 0*.*37*	*1*.*17 ± 0*.*37*	*0*.*32 ± 0*.*08*	*0*.*11 ± 0*.*04*	*0*.*05 ± 0*.*02*	*0*.*03 ± 0*.*01*

LCModel estimated relative GABA concentrations to total Cr (tCr) with Cramér–Rao lower bound (CRLB) for the sLASER and MEGA-sLASER. SNR was measured using the peak amplitude of the total NAA peak at 2 ppm and the standard deviation of the white noise area in the 7 to 9 ppm range These metabolic concentrations are directly measured values by LCModel before correcting for T_2_-relaxation. Absolute uncertainties were calculated by multiplying the relative CRLB from the estimated concentration [60]. The measured value was expressed as mean ± SD.

**Fig 3:**
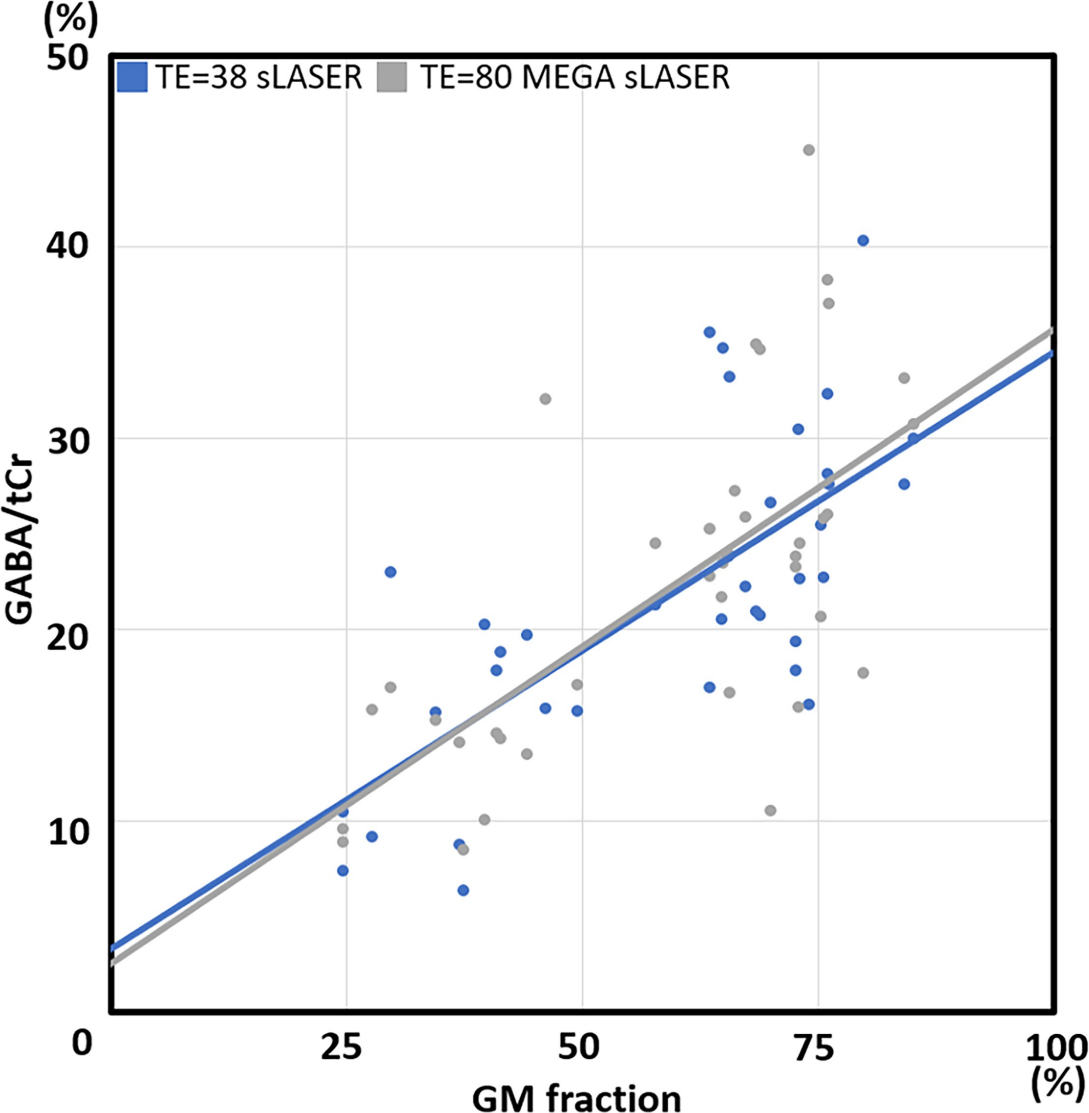
Correlation between GABA/total Cr(tCR) and gray matter (GM) volume fraction in the spectroscopy voxel. Spectroscopy signal was measured by two different approaches (blue: echo time (TE) = 38 semi-LASER (sLASER), gray: TE = 80 MEGA-sLASER) across all examined voxels after the T_2_ correction. GABA concentration was in directly proportional to the GM tissue fraction. Linear regression lines of these two methods are almost identical. Equations of the linear regression lines were *y*_*TE* = 38 *sLASER*_ = 29.27*x* + 4.68 and *y*_*TE* = 80 *MEGA*−*sLASER*_ = 33.18*x* + 2.54, unit (%).

Fig 3 shows the correlation between GABA/tCr and GM volume fraction in the spectroscopy voxel estimated with the two different acquisitions after T_2_ correction between sLASER (TE = 38 ms) and MEGA-sLASER (TE = 80 ms). We found a linear correlation between the GABA/tCr concentration ratio and GM tissue distribution (linear regression line: *y*_*TE* = 38 *sLASER*_ = 29.27*x* + 4.68 and *y*_*TE* = 80 *MEGA*−*sLASER*_ = 33.18*x* + 2.54, %). In addition, these regression lines were almost identical for the two methods. This result shows a large dependence of measured GABA concentration on GM volume fraction, independent of the two acquisition methods. On the assumption that CSF does not contain significant quantities of metabolites [64], estimated percentage GABA distributions in the GM and WM using the linear regression lines were (GM%: WM%) 86%: 14% for sLASER and 93%: 7% for MEGA-sLASER, very much in line with the literature values for the relative distribution between GM and WM [65].

**Fig 4:**
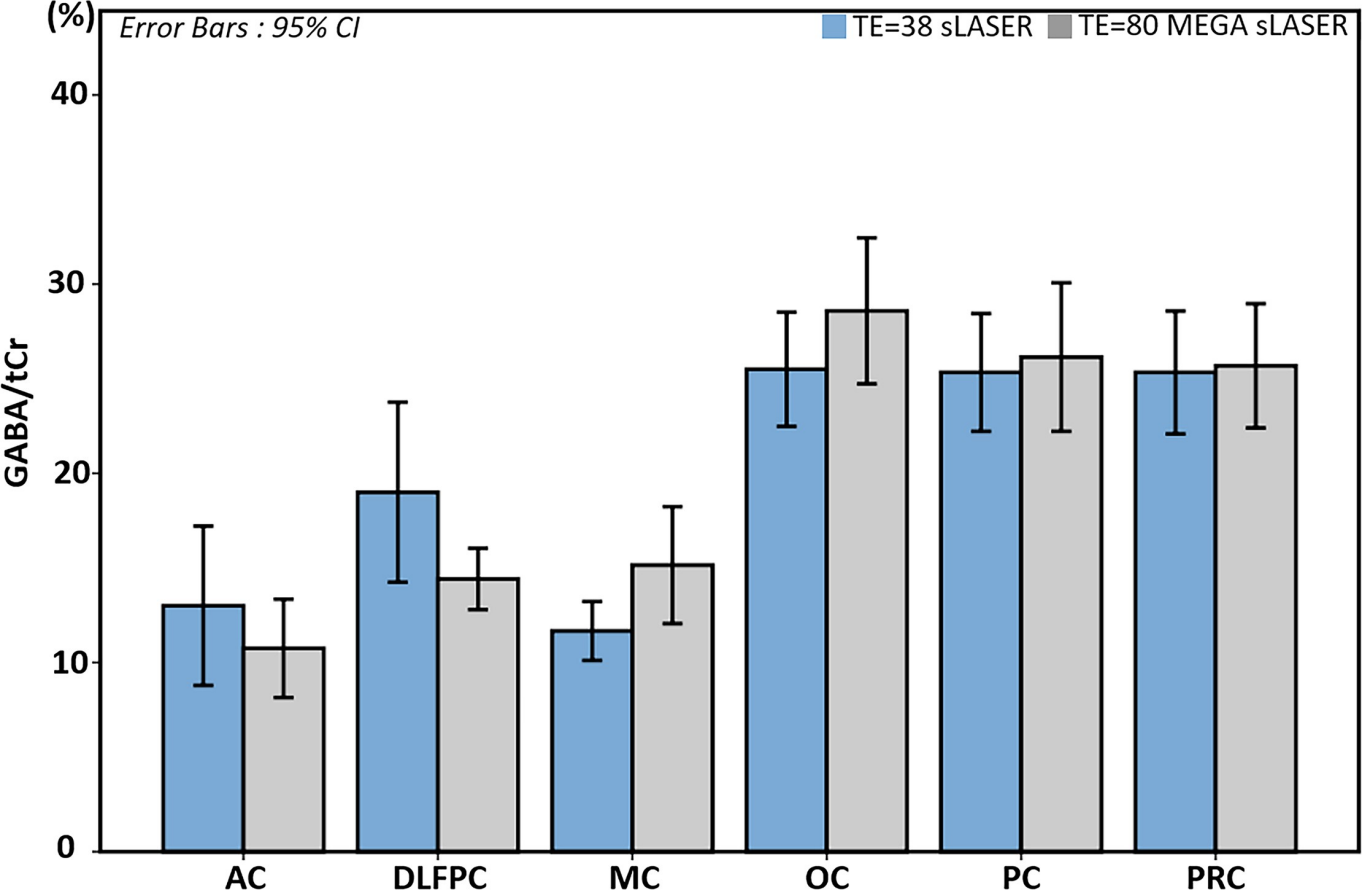
GABA/ total Cr(tCr) comparisons after considering the T_2_ relaxation times of GABA and tCr. T_2_ correction factor for echo time (TE) = 80 MEGA-semi-LASER was 1.488 for GABA/tCr. After applying this factor, we found no statistically significant differences between group means across acquisition methods determined by a one-way ANOVA. Error bars indicate 95% confidence intervals.

We also found similar GABA to tCr ratios acquired with the two methods for each voxel (Fig 4A). Error bars indicate 95% confidence intervals. Regional GABA concentrations showed statistically significant differences between group means as determined by a one-way ANOVA (F(5,36) = 0.302, p = 0.019) for the sLASER method, and (F(5,36) = 6.015, p < 0.001) for the GABA editing method. OC, PC, and PRC showed similarly high mean GABA/tCr ratios as compared with the other three regions: AC, DLPFC, and MC.

**Fig 5:**
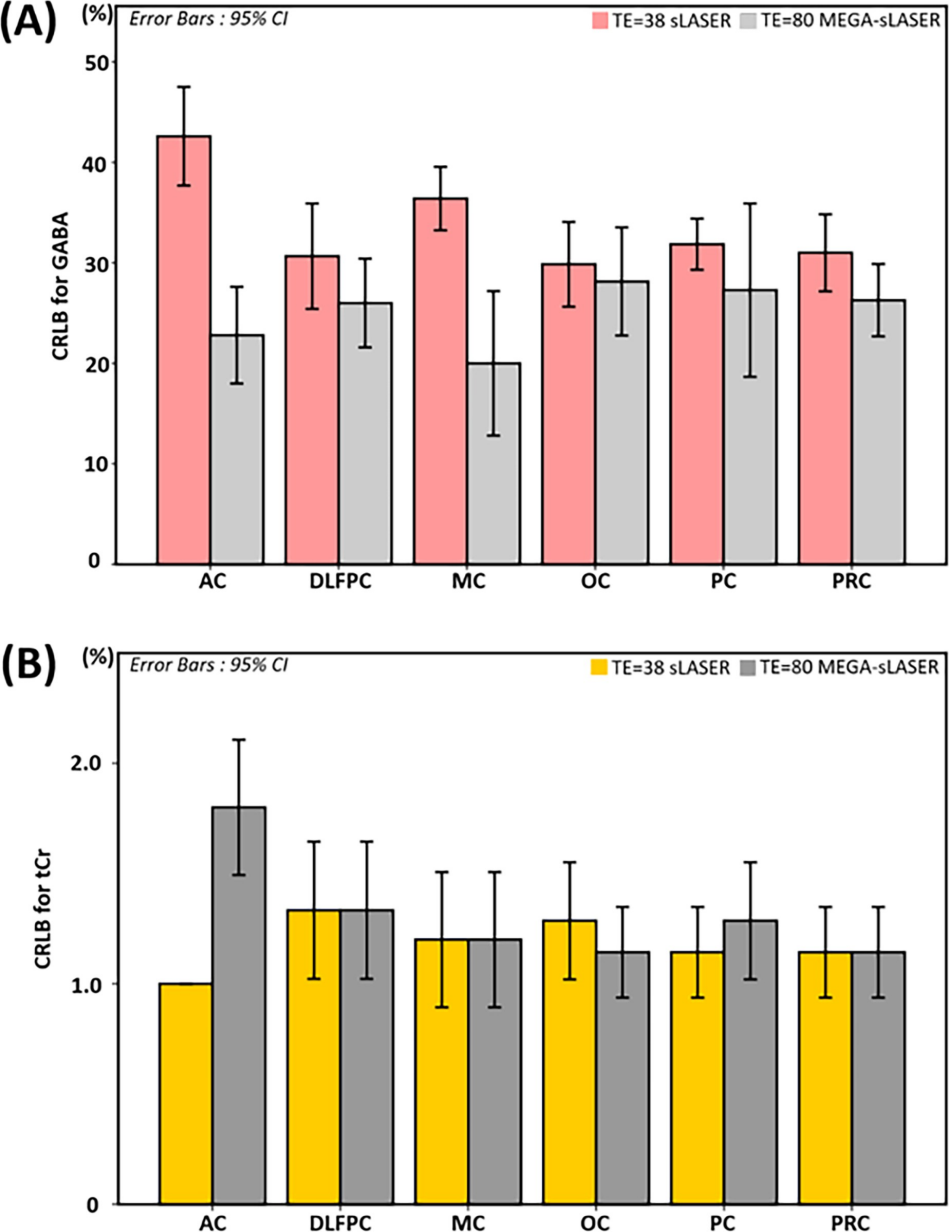
Cramér–Rao lower bound (CRLB) comparison. This figure compares CRLBs of (a) GABA and (b) total Cr (tCr) between semi-LASER (sLASER) and MEGA-sLASER for the six brain regions examined. Error bars indicate 95% confidence intervals, in accordance with Table 1.

Fig 5 shows CRLB comparisons of GABA (A) and tCr (B), estimated by the two different acquisition strategies. For both metabolites, the MEGA-editing approach showed slightly better spectral fitting quality than that of the non-editing acquisition approach according to CRLB for all regions examined. Error bars also indicate 95% confidence intervals.

**Fig 6:**
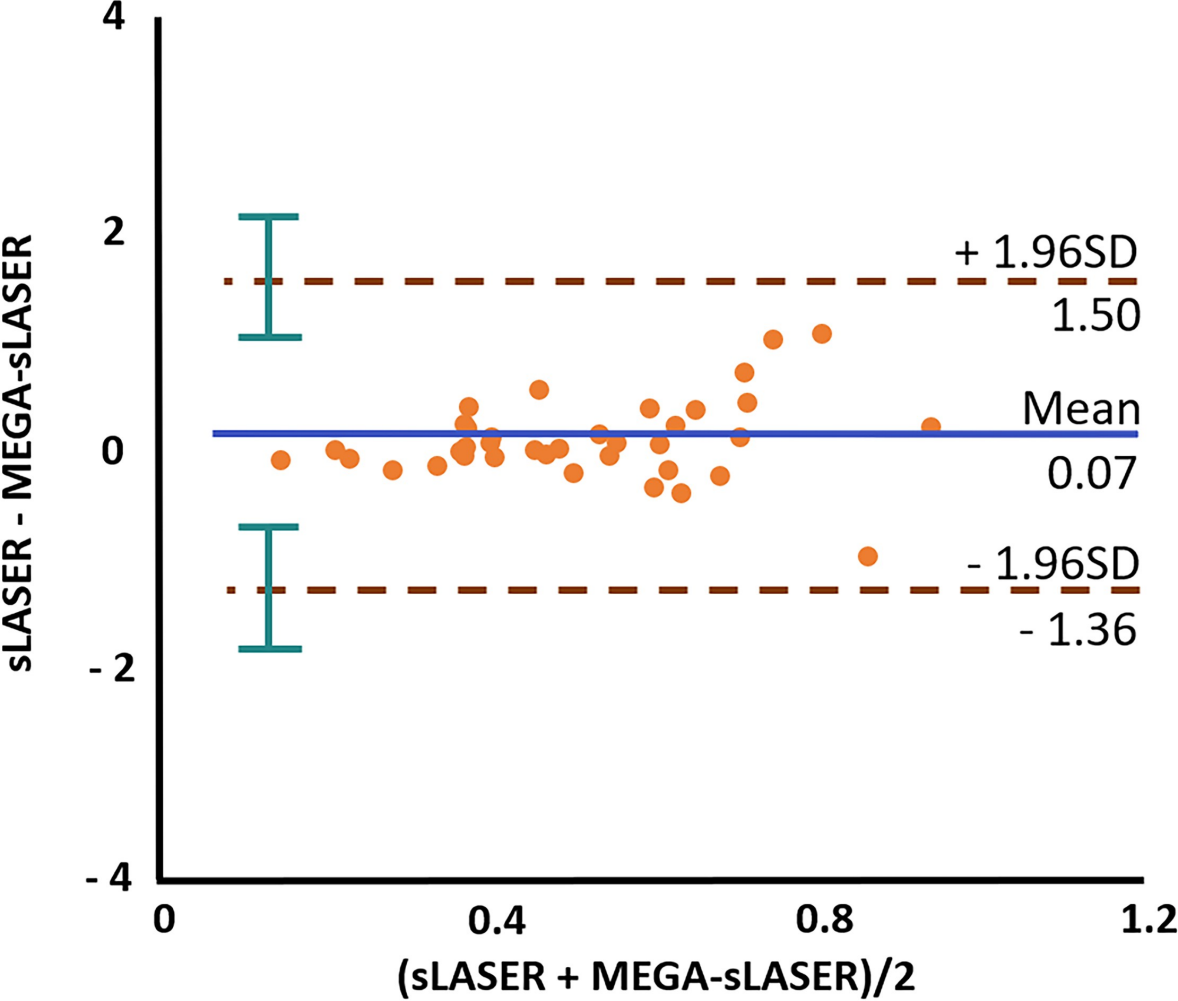
Bland-Altman plot for GABA. Bland-Altman plot of the GABA concentration from the interleaved measurements using the semi-LASER (sLASER) and MEGA-sLASER approaches afterthe LCModel analysis. The blue horizontal line shows a mean value and two brown lines show ± 1.96 * SD with 95% confidence intervals (green) across all measurements.

The Bland-Altman plot in Fig 6 shows the mean bias ± SD with 95% confidence intervals between the sLASER and MEGA-sLASER results as 0.06 ± 0.73 (blue line), and the limits of agreement were −1.36 and 1.50 (brown lines).

**Fig 7:**
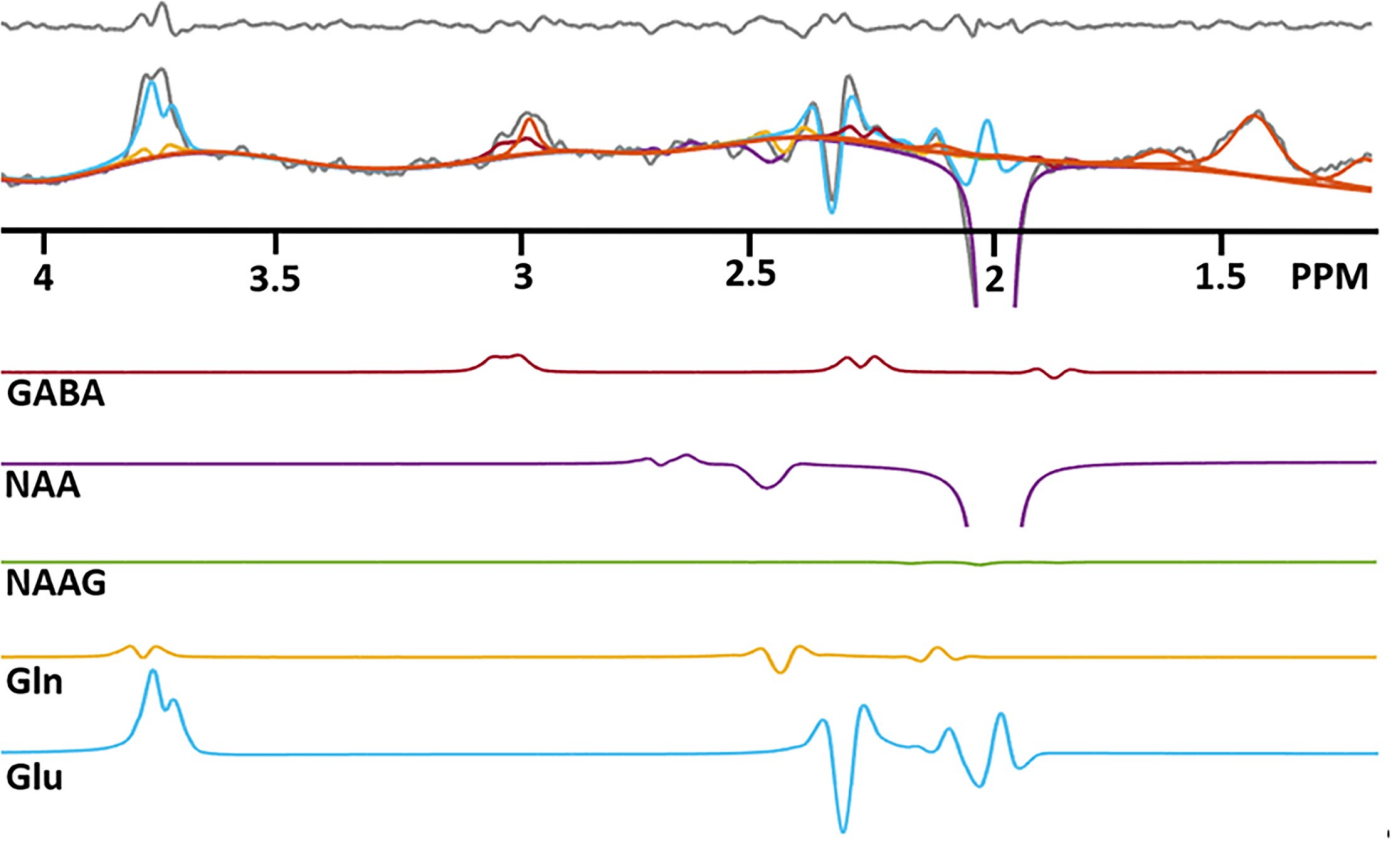
One example of GABA edited spectrum (gray) with metabolite fitting lines. GABA (red), NAAG (green), NAA (purple), Gln (yellow), Glu (blue), Glu (blue) and macromolecule (MM: orange), which were estimated from LCModel. Top panel indicates residual, which is a difference between *in-vivo* and fitting lines. Directly observable GABA-^2^CH_2_ resonance at 2.28 ppm plays an important role in the GABA quantification with spectral fitting. GABA estimation is limited by the 2.28 peak, which is the true GABA. It prevents over-estimating GABA by co-edited macromolecule. Note that the bottom part of the NAA signal is truncated in this figure.

One example of the GABA edited spectrum (gray) with metabolite fitting lines: GABA (red), NAAG (green), NAA (purple), Gln (yellow) and Glu (blue), which were estimated from LCModel is shown in Fig 7. GABA estimation is clearly to a large degree determined by the fit to the 2.28 ppm peak. Pure GABA accounts for 47.21 ± 17.04 percent of the total 3 ppm signal, calculated as the ratio of the LCModel GABA fitted line at 3 ppm to the total measured 3 ppm peak averaged over all subjects.

## Discussion

The interleaved sequence, which we implemented in this study, provides simultaneous MEGA-editing and non-editing acquisitions in a single sequence. By acquiring concurrently, the interleaved acquisition balances any instability across the two techniques. Therefore, we could avoid potential problems of registration, motion, B_1_-inhomogeneity or different shim quality that may have occurred if we would have acquired sequentially. It is widely known that adiabatic RF pulse based sLASER sequence has the advantage of the less level of CSDE in comparison of the PRESS sequence at ultrahigh field [43] owing to the use of adiabatic refocusing pulses. The sequence used in this study had a maximum CSDE of 8.4% [66].

After applying the T_2_ correction factor, which compensates the different TEs, the GABA/tCr ratios for the two acquisition techniques did not differ significantly. From the fact, almost all difference values are within the confidence limits, and the p-value is greater than 0.05, we conclude that there was no significant difference between the GABA acquisition methods (See Fig 6). In addition, the uniform distribution of difference values shows that there is no systematic bias between the two methods (calculated bias = 0.01). Judging from results of the linear regression lines (see Fig 3) and the Bland-Altman analysis (see Fig 6), the two different approaches showed similar accuracy in terms of the GABA estimation across the brain regions in combination with proper compensation steps.

### Regional GABA concentration

Even though the main purpose of this study was to compare the two methodological approaches in combination with LCModel analysis, we were also able to compare GABA concentrations across different brain regions. As we mentioned in the Introduction, it is widely known that GABA is mostly concentrated in GM (approximately 90% in GM and 10% in WM). Not only spectroscopy studies [30, 65, 67] but also an *ex-vivo* study [68] obtained similar GABA distribution ratios between GM and WM. Chen, *et al*. [69] reported that GABA concentration linearly increased with GM volume fraction for one of the brain regions we also studied (AC). In our result, as we can see in Fig 3, GABA concentrations were largely driven by the GM tissue fraction. In addition, estimated GABA distribution ratios in the GM and WM by the linear regression lines are in close agreement with the literature value. This result confirms that GABA is heavily concentrated in GM, and highlights the importance of the tissue volume correction for GABA quantification [70].

In addition to the current study, other studies, which investigated regional GABA concentration differences while accounting for tissue volume variations, found differences in the regional GABA level. However, there is some inconsistency in the literature. For instance, Ganji, *et al*. [30] reported higher concentrations of GABA in the frontal lobe than in the OC at using PRESS (TE = 92 ms) at 7 T. Öngür, *et al*. [71] found higher GABA/Cr ratio in a frontal region (AC) than in an occipital region (parieto-occipital cortex) measured with the MEGA-editing method at 4 T. On the other hand, Bhagwagar, *et al*. [72] found higher GABA/Cr ratio measured with MEGA-editing in the occipital region (OC) than that in AC. Durst, *et al*. [73] also reported higher GABA in OC than in frontal lobe using 3 T MEGA-PRESS. Van der Veen, *et al*. [74] similarly found higher GABA concentration in the OC than that of the anterior area (MPFC) with the MEGA-editing method comparing two voxels in frontal and occipital lobes. Van der Veen, *et al*. [74] selected voxels that had the same GM and WM tissue volume to exclude tissue volume effects. They proposed therefore that the difference should be attributed to factors other than GM volume. The trend in our data is in accordance with that of the latter studies [72–74].

Recent molecular Imaging studies, which quantified GABA_A_ receptor using positron emission tomography (PET) with ^11^C flumazenil [75, 76], and single-photon emission computed tomography (SPECT) with ^123^I-iomazenil [77] have visualized GABA_A_ receptor densities in the human brain. These reports have shown not only that the GABA_A_ receptor is more heavily concentrated in GM, but also its density in posterior brain areas is higher than that of anterior areas. This supports our finding that GABA concentration in posterior regions such as OC, PC, and PRC showed higher GABA levels than those of anterior regions: AC, DLPFC, and MC.

### MM contamination

We found no statistically significant difference for the quantified GABA concentrations with LCModel between two acquisition techniques. Our finding could potentially be explained by one of two possibilities: The LCModel fitting result of the non-editing spectrum also contains MM contamination, or LCModel efficiently quantifies the GABA signal from the GABA edited spectrum without the interference of the co-edited MM.

LCModel estimates MM and lipid signals at each fixed chemical shift location with non-parametric spectral models including a smoothing B-spline as a baseline [24]. Because these MM and lipid signals are not only measured values but also part of the formulaic model, these quantification approaches still risk over- or under-estimating metabolic concentrations in the co-edited signal. It is quite challenging to determine from our LCModel quantification results of sLASER whether the estimation of GABA is contaminated by MM. However, we can assume the presence of MM contamination in the MEGA-sLASER spectra. In contrast to MM and lipid signals, metabolic signal quantification uses parametric spectral models, which are included in a basis set. LCModel finds metabolic concentrations by changing the amplitudes of simulated metabolic models with their lineshapes unchanged. Therefore, all resonances of GABA also play an important role in solving the optimization problem. In our spectral fitting results, we found that the directly discernible 2.28 ppm GABA signal was essential for distinguishing the GABA signal from GABA+ (See Fig 7). GABA estimation was largely determined by the size of GABA-^2^CH_2_ resonance at 2.28 ppm. Because the 2.28 ppm region is not related to the co-edited MM, the visible GABA resonance at 2.28 ppm represents an uncontaminated GABA signal. This result suggests that GABA quantification with spectral fitting is able to separate pure GABA from GABA+ provided that the 2.28 ppm GABA resonance is also recorded. Previous studies by Ganji, *et al*. [30] and Choi, *et al*. [78] also highlighted the significance of this GABA-^2^CH_2_ resonance in terms of the spectral fitting procedure.

LCModel provides a GABA-editing exclusive control file called ‘mega-press-3’ [56]. This setting excludes quantification of the baseline and MM signals assuming baseline and MM signals are identical between MEGA-on and -off spectra, and that they hence cancel in the subtraction. Since this setting mainly attempts to estimate the 3 ppm peak as GABA+, this necessarily overestimates the GABA concentration as co-edited MM will not be eliminated. Therefore, we did not use this approach.

We corrected for T_2_ relaxation using the T_2_-value of 63 ms taken from the study by Intrapiromkul, *et al*. [62]. After correction, the values obtained were similar between the two methods (See Fig 3 and Fig 4A). This consistency strongly supports the correctness of the T_2_-value used, and also that the analyses have yielded results without MM contamination. Our estimate that MM constitutes approximately 53% of the signal at 3 ppm is at the upper limit of, but not inconsistent with previous literature values 44% [15], 41–49% [79], and 52–57% [80].

### Spectral fitting quality

Since the thresholded CRLB estimation may cause a bias [60], we included all GABA and tCr results. We also compared absolute uncertainties for the GABA estimation for both acquisition methods. As a result, we found that CRLBs and absolute uncertainties of estimated GABA for the MEGA-editing method are lower than those for non-editing acquisition method for all six regions (See Table 1 and Fig 5A).

Compared with the non-editing acquisition, the GABA editing method provides a much simpler spectral pattern with only six metabolic signals present, that are relatively well separated. This explains why the spectral fitting accuracy of the MEGA editing results for estimating GABA is superior to that of the sLASER result, despite the editing method eliminating also a number of lines from the GABA spectrum. MEGA-sLASER and sLASER showed similar CRLB values for tCr (Fig 5B) which is unsurprising given that the tCr concentration of the MEGA-sLASER was estimated from the MEGA-off spectra using a non-editing acquisition. The reasonably good CRLB values of tCr for both approaches confirm that spectral quality and spectral fitting quality with LCModel were good enough for accurate quantification.

## Conclusion

This study compared GABA concentration for six different brain regions with the non-editing spectral acquisition approach and the GABA editing approach using a single interleaved sequence acquisition. The simultaneous acquisition of sLASER and MEGA-sLASER minimized differences in confounds which could have occurred in individual acquisitions. Our finding confirms that GABA is heavily concentrated in GM. Therefore, regional GM volume is an important contributor to measured GABA concentration variation. In addition, the ability to reliably measure the GABA resonance at 2.28 ppm would appear to lead to accurate estimates of GABA concentration without MM contamination. A similar concentration of GABA signal measured with the two methods for each voxel was obtained after correction for T_2_, showing that the two acquisition strategies are both reliable and have a similar accuracy to measure GABA. However, The GABA-editing approach was superior in terms of precision, as assessed by lower CRLB values.

## Supporting information

S1 File Result dataset. (XLSX)
